# Behavior Life Style Analysis for Mobile Sensory Data in Cloud Computing through MapReduce

**DOI:** 10.3390/s141122001

**Published:** 2014-11-20

**Authors:** Shujaat Hussain, Jae Hun Bang, Manhyung Han, Muhammad Idris Ahmed, Muhammad Bilal Amin, Sungyoung Lee, Chris Nugent, Sally McClean, Bryan Scotney, Gerard Parr

**Affiliations:** 1 Department of Computer Engineering, Kyung Hee University, Suwon 446-701, Korea; E-Mails: shujaat.hussain@oslab.khu.ac.kr (S.H.); jhb@oslab.khu.ac.kr (J.H.B); smiley@oslab.khu.ac.kr (M.H); idris@oslab.khu.ac.kr (M.I.A.); mbilalamin@oslab.khu.ac.kr (M.B.A.); 2 School of Computing and Information Engineering, University of Ulster, Newtownabbey, Co. Antrim, BT38 0QB, UK; E-Mail: cd.nugent@ulster.ac.uk; 3 School of Computing and Information Engineering, University of Ulster, Coleraine, Co. Londonderry, BT52 1SA, UK; E-Mails: si.mcclean@ulster.ac.uk (S.M.); bw.scotney@ulster.ac.uk (B.S.); gp.parr@ulster.ac.uk (G.P.)

**Keywords:** activity recognition, mobile cloud, MapReduce, behavior analysis, big data

## Abstract

Cloud computing has revolutionized healthcare in today's world as it can be seamlessly integrated into a mobile application and sensor devices. The sensory data is then transferred from these devices to the public and private clouds. In this paper, a hybrid and distributed environment is built which is capable of collecting data from the mobile phone application and store it in the cloud. We developed an activity recognition application and transfer the data to the cloud for further processing. Big data technology Hadoop MapReduce is employed to analyze the data and create user timeline of user's activities. These activities are visualized to find useful health analytics and trends. In this paper a big data solution is proposed to analyze the sensory data and give insights into user behavior and lifestyle trends.

## 1. Introduction

Activity recognition, remote monitoring and healthcare services provisioning are gaining more importance with active lifestyle of users and development of sophisticated technology. There are many obstacles associated with activity recognition and automatic monitoring due to diversity of devices. Existing procedures for collecting user's vital signs are tedious, invasive and requires a lot of efforts to get and analyzed. Nowadays mobile devices are one of the main platforms for getting as well as communicating the health information. The integration of healthcare applications with smartphones can lead to efficient and higher quality of health care [1]. However, there are many challenges faced by the smartphones and mobile devices with regard to computation and energy consumption [2]. Those limitations must be addressed when developing mobile applications. Cloud Computing has recently become a popular paradigm for leveraging mobile phone applications [3]. As a result, cloud based services are more frequently being used as part of smartphone based applications. Moving data and computation from mobile devices to large data centers makes sense as it enables users to obtain a better experience from a range of perspectives, most notably being the increase of service performance. The latest trend is to build mobile applications in which the data is transferred and seamlessly available to laptops and computers in addition to applications such as dropbox [4] and icloud [5].

According to latest research, the market for cloud based mobile applications will reach $9.5 billion by 2014 [6]. Cloud computing services are becoming integrated into many diverse types of mobile applications varying from games and social networking to health and social care applications. However there are still many point of views related to mobile cloud computing. One viewpoint is that both data storage and data processing for mobile applications should be performed outside of the mobile device [7]. An alternative viewpoint is to connect the network of mobile devices in a peer to peer network for resource sharing [3]. In our current work the former point of view is followed mainly due to the fact that mobile cloud computing is an extension of cloud computing and is termed as an ad-hoc infrastructure [8]. A further advantage of the concept is that mobile cloud applications are not limited to a certain device or operating system. Based on this smartphones are considered as the mobile device within our work as they are easily connected to the internet through wireless and 3G and 4G networks. Although mobile cloud can offer a number of advantage as previously outlined it also brings a set of problems and challenges with it. Many problems occur related to the diversity of network conditions (low bandwidth), disconnection, and limited power [2].

This study focuses on user's activity recognition in different location which can help in recommending healthy life style for the user. The activities user perform shows a great insight in user's choices and behavior. By analyzing these activities, user's lifestyle can be identified and recommendations can also be made to improve his health. Mostly in the literature activity recognition is not coupled with the user's behavior and his lifestyle. The data gathered from activity recognition is very insightful and important for a peek into user lifestyle. The data gathered also makes it very tricky to be analyzed completely because of limited processing power and battery time.

The current solutions are manual and slow due to lack of real time data collection which results in slow monitoring and diagnosis. With this in mind a mobile application is needed which will be optimal in terms of energy and processing power and does not drain the battery time. The study focuses on personalized activity recognition for life care by using different mobile sensors through feature extraction and classification methods. One of the main focus is to collect the data in the cloud and understanding the user profile and his activities. Hadoop [9] is used which is an open source implementation of MapReduce. The training data was collected and the implementation for our algorithm was done to find user behavior which are used for recommendations. Our technique achieved 94% accuracy while detecting user activity in different locations such as work, home and outdoor. The technique also saves the activity data and created a life log repository on the cloud where the logs can be processed to predict user behavior. The application also sends the battery consumption information to know how the mobile application affects the smart phone battery.

The remainder of the paper is organized as follows. Section II briefly reviews related work in the area of activity recognition and mobile cloud computing. Section III proposes a framework for the mobile application and the mobile cloud services and storage and Section IV explains the implementation and results and Conclusions and Future Work are presented in Section V.

## 2. Related Work

The smartphones nowadays have built-in sensors which are highly effective for activity and context identification. In the research work presented in [10], a smartphone was used to identify walking and running [11] whereas GPS data was used to recognize transport and commuting.

‘Nike + iPod’ [12] initiated by Nike, logs user well-being activities such as running, jogging, and gym activities via the Nike+ hardware device paired with Apple's iPhone or iPod. Activity data is subsequently published over Nike's portal [13], which provides data visualization services and data persistence services. ‘MapMyRun’ [14], is a similar application that keeps track of user's workout activities and nutrition intake with intuitive visualizations and track mapping services. Activity data gathered from a smartphone can be stored over the MapMyRun Portal [14] or exported as log files to be sync with cloud storage services like dropbox [4].

Maintaining log files for activities is turning out to be very important as it contains vital information about our well being. These logs can vary from our daily life activities to our workout and exercise activities. Most of the available life-logging applications are focused on well being and workout tracking. Life-log data recorded by the smartphones provide improved activity tracking by utilizing the built-in sensors and GPS capabilities of the phone. A novel feature selection algorithm is used for accelerometer classification [15] and it utilizes multimodal sensor data from accelerometer, audio, GPS, and Wi-Fi. Another approach based on this technique takes the context information and prompts the user for an activity label [16]. This label and the sensory data is saved and stored in the cloud. Another smartphone based hierarchical approach is used for activity modeling and real time activity recognition [17].

These applications utilize cloud and web for the persistence of activity data. This data is used as the basis for improved visualization over the web and smartphone, and can also be used for expert analysis such as physicians and trainers.

Cloud computing has introduced a new revolution in the development of the internet. The rapid rise of cloud computing and mobile computing has started a new computing paradigm that is mobile cloud computing. Mobile cloud computing has, however, a set of challenges once integrated into a mobile application with a cloud service. There have been many elastic models for mobile applications as the mobile application is launched inside the mobile device, however, later the processing or data is migrated to the cloud. Research in mobile cloud computing has ranges from topics considering energy saving, data management to migration, social networks, and healthcare.

The potential of applying mobile cloud computing for purposes of monitoring healthcare has the potential of minimizing costs of traditional health care treatment. Monitoring patients and accessing medical records easily at all times is a clear advantage. In addition, taking action with some intelligent emergency management system when the patient has been identified as being in distress is a further advantage. The concept of the Health cloud [18], is a prototype which utilizes the public Amazon cloud to manage patient records and relevant medical images. The Project has developed an android application for viewing JPEG2000 standard images with image annotation exploiting the multi-touch functions of the Android OS. The mobile device is now an essential part of the distributed architecture [19] and analysis of sensory data to determine human activities are done using MapReduce and many studies are now using big data technology for extracting context out of sensory data.

## 3. Proposed Framework

**Figure 1.**
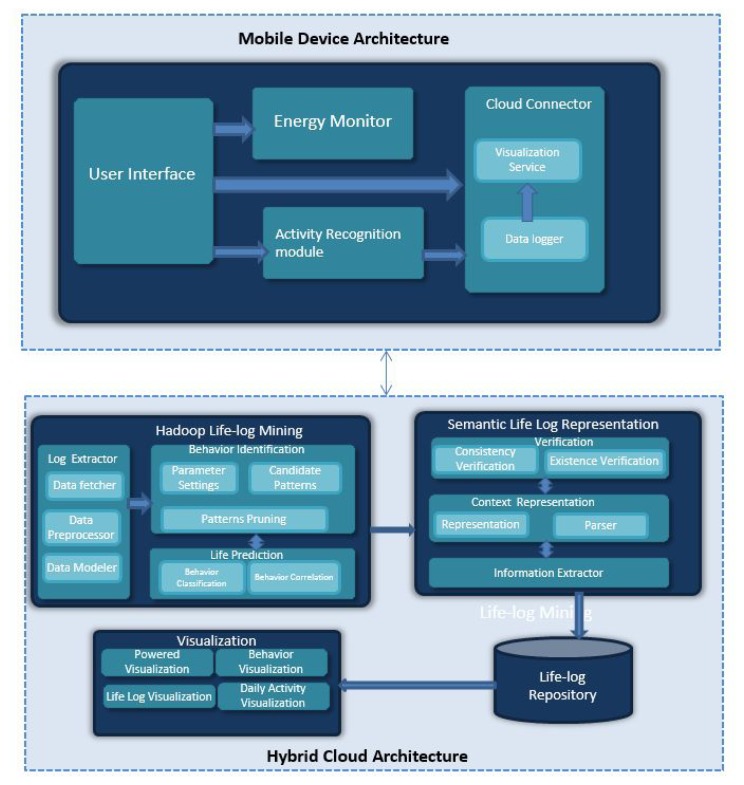
Main framework Architecture.

In this section the proposed system is discussed and its components are explained in detail. It consists of two main parts, *i.e.*, the mobile application and the cloud computing storage and processing module in Hadoop as shown in Figure 1. The mobile application uses different embedded sensors of smartphone.

The mobile application consists of three main modules, *i.e.*, activity recognition module, energy monitor and cloud connector. Activity recognition is the core component which uses different sensors to recognize nine contexts with three different activities in three different locations. Naive Bayes classifier [20] is used for recognizing human activities. If the recorded activity of the user is matched to the model saved, it is chosen by the algorithm.

The application is designed in a way where user interface passes the sensory data after communication with the energy monitor and activity recognition modules. The data passed consists of energy measurements and activity labels as well as sensory logs. The cloud connector sends the compressed data to the cloud component. The cloud component is responsible for behavior analysis and advanced visualization.

The proposed system benefits from the advantages of cloud computing through big data technology. Storing the data in the cloud helps reduce costs that are usually incurred to store data on local servers and replicate this data on backup servers. Ubiquitous access of data from the public cloud allows multiple devices (including smart phones, laptops and personal computers) to access the data and services instantaneously over the Internet. In this way resource management and making optimal decision based on the information on the cloud through visualization and mining is also achieved.

### 3.1. Mobile Device Architecture

The mobile application consists of the user interface. Through the user interface there is a configurable energy monitor in which the battery level threshold is defined to shut down the application.

The user is given a prompt on the smartphone showing that battery levels are very low and the mobile application is going to shut down. Different embedded sensors are used for activity recognition module. The cloud connector is connected to the user interface which takes the data from the activity recognition module as well as energy monitor to the cloud for further processing. It also fetches the data from the cloud whenever visualization service is called. The visualization service gives the user indication about his activities and their frequency and duration. The activity recognition module developed in [15] is utilized.

This system has specifically used Hidden Markov Model (HMM) [21] for audio classification. HMM algorithm is used for training and learning the acoustic signals to recognize the bus sound. In case of riding the bus activity, audio sensor can be one of the best resource to classify the activity. In order to work in the real-time environment it is trained over the server machine and classification is performed inside the mobile environment. The more details can be found in our referred paper [15]. We utilized Gaussian Mixture Model (GMM) for the classification of accelerometer data and it works well in the ‘limited environment. We observed that GMM does not perform well when number of contexts increased. We introduce Naive Bayes classifier in this paper that leads to the light-weight and works well completely in the smartphone environment.

**Figure 2.**
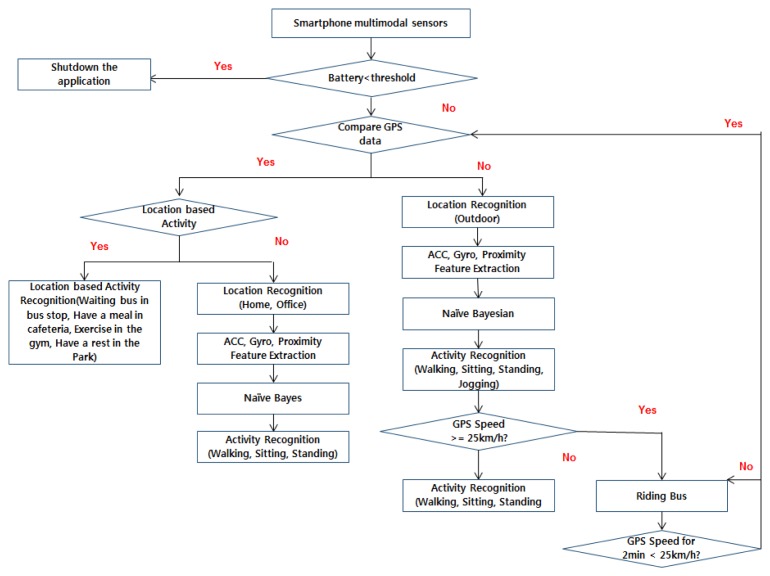
Flow Chart for activity recognition.

This model uses sensor data from accelerometer, GPS and Wi-Fi to identify activities like walking, sitting and standing mainly but are extended to more activities like (jogging, resting in park, waiting at bus stop, having lunch in cafeteria, exercising in gym and riding a bus) in outdoor locations. The mobile application makes use of several sensors, *i.e.*, accelerometer, gyroscope, proximity and light sensors to identify different contexts. The overall flow of the application is given in Figure 2.

In pre-processing setup, we divide the time-series sensory data over the 3 sec window. We adopt no overlapping window method to chunk the signals. In the next step we extract the time and frequency domain features. The Naive Bayes classifier is used as our core algorithm for the accelerometer sensor data to recognizing the user activities. We construct the model to distinguish the user performed activities at different locations. If the performed user activity is similar to the pre-constructed activity model, that activity is chosen as a recognized activity. Naive Bayes is chosen for its faster modeling time and work well inside the smartphone environment. The Naive Bayes classifier assumes that sensory data D_1_ to D*_n_* have possibilities relating to an independent class C. The probability of C after the sample data D_1_…..D*_n_* are collected is p(C | D_1_,…., D*_n_*) which is referred to as the posteriori probability. In order to calculate p(C | D_1_, ….., D*_n_*),(D_1_,…., D*_n_*) and p(C) are required. These can be estimated from training data and are referred to as the boundary probability. The implementation is being done in MapReduce displayed in Algorithm 1. We use the map function to get the the frequency of all the activities and for the sensory data we calculate the point and activity. This is passed as an serialized object to the reduce function. The reduce function then deserialize the object and calculates the probability of the activities shown in Algorithm 2. By using Bayes theorem a posteriori probability is defined by Equation (1):

(1) $$\mathrm{Pr}(C|D_{1},D_{2},\ldots ..D_{n})=\frac{\mathrm{Pr}(D_{1},D_{2},\ldots ..D_{n})|C)\mathrm{Pr}(C)}{\mathrm{Pr}(D_{1},D_{2},\ldots ..D_{n})}$$

The locations in the application in this case are home and office. From the sensors, *i.e.*, the accelerometer, Gyroscope, GPS, Proximity the data is collected and then feature extraction is used as well as Naive Bayes to find out the current activity. If the current GPS data is unfamiliar, then the user is in outdoor location. If the GPS speed is greater than 25 km, then the user is in a bus/car. So when the speed is less than 25 km then the activity recognition is again started from comparing the GPS data.


**Algorithm 1** Bayes Map Function.
 **Input:** data sets **Output:** Labeled Objects *freq* ←sum(activity) **for**
*D_i_ IN D_n_*
**do**  *object.point* ⇐get(D*_i_*)  *object.activity* ⇐get(activity)  *serObj* ⇐serialize(D*_i_*)  collectoutput(activity,serObj) **end for**



**Algorithm 2** Bayes Reduce Function.
 **Input:** Labeled Objects **Output:** Classified Object **for**
*activity_i_ IN activities*
**do**  *object* ⇐deSerialize(serObj)  **for**
*activity_j_ IN object*
**do**   *object* ⇐deSerialize(serObj)   *prob_j_* ⇐calculateProbability(*activity_j_*)   *prob_i_* ⇐calculateProbability(*activity_i_*)   **if**
*prob_i_* > *prob_j_*
**then**    *prob_i_* ⇐calculateProbability(*activity_i_*)    *activity* ⇐assign(*activity_i_*)   **end if**  **end for** **end for**


The user is located in outdoor area if the GPS signal is available and strong enough. The system recognizes the activity and then the location for differentiating indoors (Home and Office) and outdoors. If the user is at an unknown location, the system tries to recognize the activity among outdoor activities, *i.e.*, walking, sitting, standing, jogging, rest in park, waiting at bus stop, having lunch in cafeteria, exercising in gym and riding a bus).

One of the main function of our mobile application is activity recognition during different time of the day. In total, nine activities are recognized for outdoor locations. Furthermore we have three different activities for home and office locations. The activities are the same; however, the meaning of the activity in a specific location is different. For instance, if the system recognizes ‘sitting’ at the ‘office’, it means the user is ‘working’ or if user is undertaking the same activity at ‘home’, it would be considered as ‘taking a rest’. In our current implementation, the data is stored on the device and the system generates an activity model from the collected training dataset and stores the results.

We have evaluated the activity recognizer with three different activities (Standing, Sitting and Walking) at three different locations (Home, Office and Outdoor). Additionally for outdoor locations we have jogging, rest in park, waiting at bus stop, having lunch in cafeteria, exercising in gym and riding a bus. Home and Office are specific locations based on GPS data. In our current approach, each activity is trained with specific location and acceleration, so recognizable activities are treated as different even if the activity is same. The activity recognition is done in a robust way and regardless of the orientation of the smartphone. The smartphone was placed in the trousers' front pocket which is the most common choice of most of the users. As the activities depend on the motion patterns of the legs. We collected the data in a six week period without any supervision and particular positioning of the smartphone. We use accelerometer sensor for recognizing “Sitting” event. We extract value of mean and standard deviation from each of 3-axis accelerometer sensor.

The accelerometer sensor data sampled at 50 Hz is converted into time and frequency domain features, which is used as input to Naive Bayes classifier. The pre-processing component collects sensing data from multimodal sensors after a particular time interval and brings into an acceptable format for fast data processing and passing it to the cloud. GPS is also extensively used to gather the walking, sitting, and standing activities.

The following features are considered:

- Time domain features: standard deviation, mean crossing rate, Pearson correlation coefficients
- Frequency domain features [22]
- Linear Predictive Coding (LPC) features [23]

In time domain features, the selected features were standard deviation value, mean crossing rate and XY correlation. For frequency domain features we used over spectral energy, spectral sub-band 1 energy and spectral sub-band 2 energy. For linear Predictive Coding (LPC) we used LPC coefficient 1, LPC coefficient, LPC coefficient 3 and LPC estimation error.

**Figure 3.**
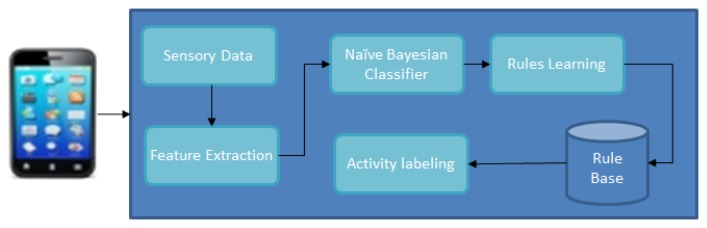
Architecture for training and testing data.

The activity recognition architecture is shown in Figure 3. The smartphone data is sent to feature extraction to extract representative features for identifying the activities. Then the training phase starts in which learning and labeling the performed activities is done and later to recognize the activity in the testing phase. We classify contexts, such as in a bus, park, or meeting place, through an audio sensor audio data. For the audio classification, HMM algorithm is used for training and testing audio.

### 3.2. Hybrid Cloud Architecture

The sections below discuss in detail the components of hybrid cloud architecture shown in Figure 1 and the role of Hadoop MapReduce in life log mining. In this section we identify user behavior through MapReduce after activity recognition is done in mobile device architecture. The semantics of the activity recognition is extracted and behavior classification is done through correlation.

#### 3.2.1. Hadoop Life Log Module

Hadoop is a cloud computing platform and an open source implementation of MapReduce programming model. In a MapReduce job there are three phases, *i.e.*, map, copy and reduce.

As all the sensor data(Accelerometer, Gyroscope, GPS, Proximity) is recorded for future use and provides logging application for Android Smartphone. The application can collect all sensors data from smartphone that is Accelerometer, Gyroscope, Orientation, GPS, Light and Proximity. The life log module is capable of mining life-logs and hence be able to evaluate a user's behavior based on life conditions and constraints. This component analyzes the user's behavior relating to a set of daily activities to assist them in daily lives according to their interests.

In Map Phase shown in Algorithm 3 the activities are differentiated and the times with respect to their locations. The log extractor is be done in the map phase and the logs is be structured and preprocessed in it. The timestamps and 3 axis are extracted from the data. All the data is parsed line by line in the default mapper of Hadoop. A location class and its object is created. All the information is binded to it and serialized. The serialized object is the value and timestamp is sent as a key to the reducer shown in Algorithm 4.

These activities are then co-related with the timestamps, spatial-temporal activity information in order to map users' life events and obtain a timeline and their frequency of occurrence in reduce phase. This helps in identifying the behavior identification. The Behavior identification identifies the frequent and regular behavior of users from their previously recorded profiles. Life prediction classifies the user behavior for future predictions and long term recommendations. The parameters needed for Hadoop are the number of mappers and reducers, file size, patterns that are needed for pruning of unnecessary information as well as the different classifiers that were used in detecting the activity.


**Algorithm 3** Map Algorithm for frequency of Activity.
 **Input:** Set of Sensory data **Output:** TimeStamp as key, coordinates as value *timestamp* ←extractTime(value) **for**
*value IN values*
**do**  *timestamp* ⇐extractTime(value)  *object.xaxis* ⇐extractX(value)  *object.yaxis* ⇐extractY(value)  *object.zaxis* ⇐extractZ(value)  *serObj* ⇐serialize(obj)  collectoutput(timestamp,serObj) **end for**



**Algorithm 4** Reduce Algorithm for frequency of Activity.
 **Input:** TimeStamp as key, coordinates as value **Output:** Similar time lines and frequency of location visits *timestamp* ⇐extractTime(value) **for**
*value in values*
**do**  *object* ⇐deSerialize(serObj)  *loc* ⇐extractLocation(object)  matchLoc(loc)  createTimeline(timestamp,loc)  *frequency* ⇐aggregateSimilarTimelines(value) **end for**


#### 3.2.2. Semantic Life Log Representation

The Context Extractor extracts relevant activity information and then the context information is logged in the life log repository in compliance with the Context Representation Model. Consistency Verification verifies the consistency of the represented information for both consistency and existence and following verification, the parser parses the incoming information and then saves it based on the format specified in the life log repository.

The consistency of the data is verified through normalization to remove redundancies. There can be many inconsistencies while updating the life log so inconsistency is checked for both semantic errors and syntactic errors. Before updating, functional evaluation is done whether the update is feasible or not. Rules are validated and if it does not conform to functionality the data is tuned for validating it and the information extractor is an interface which connects to the life-log repository.

The contextual information is converted into the ontology after context verification and logging. The context information logged in life log repository is needed for behavior analysis. The context information has long term and short term patterns of the user which is gathered through smart phone (activity recognition and locations). The purpose of this module is to fuse the daily life context information of the user coming from various sources and build user profile based on the emerging context information. A logically structured and semantically sound ontology based on life log repository is created for logging the user context information.

**Figure 4.**
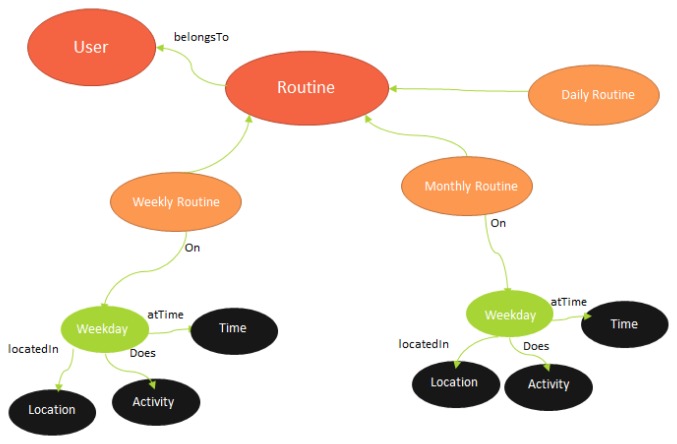
Structure of User Routine ontology.

Figure 4 illustrates the structure of user routine ontology. A routine is usually repetitive or recursive. It can be a daily, weekly or monthly routine. As our activity recognition model has high importance on the outdoor locations and different user key locations, so a routine's most important characteristics are sequence of user locations, activities and the time interval user spends doing an activity at a location. This structure shows spatial and temporal attributes of a user routine.

A user can have many activities. We are catering the activities happening on weekdays as it shows the most relevant routines and is quite reflective of user behavior which is integral to our architecture. The time interval, locations and activities are the most important routine items for user behavior which is explained more in Section 4.

There are many reasoning engines working over Web Ontology Language (OWL) format data, such as Racer [24], SWI-Prolog [25], Pellet [26], *etc.* A list of OWL implementations can be found at [27]. In our current implementation, we use Jena library [28] to handle OWL format context data and ontologies. Therefore we exploit the Jena generic rule reasoner [28] to make inference over our context data.

It provides analysis and recommendation applications over the life log as a computation is required for data mining on the cloud. It provides a visualization Service and provide intuitive graphs and statistics for better user understanding of the life log and user behavior. The approach achieves accurate life analysis pattern, improved behavior extraction and improved recommendations based on behavior analysis and life analysis pattern.

#### 3.2.3. Visualization

The visualization component within the architecture provide users with understandable visualization of the life log and user behavior based on data relayed from the mobile device.

The activity data is collected from the smartphone and labeled with respect to the activity with additional attributes like location, time and day. These attributes adds to the context of the situation. The lifelog repository also includes user's social information like tweets and restaurant check-ins as well as calories included in different food items.

The visualization component has the potential to help in identifying anything abnormal relating to the user's health and behavior. The life log and behavior visualization present graphs relating to the user's behavior in relation to a certain time period from the repository.

The life log visualization highlights the user activity in terms of most popular activity and patterns. It indicates the current situation in the user's life, where he eats and spends his time and what his favorite activity is.

**Figure 5.**
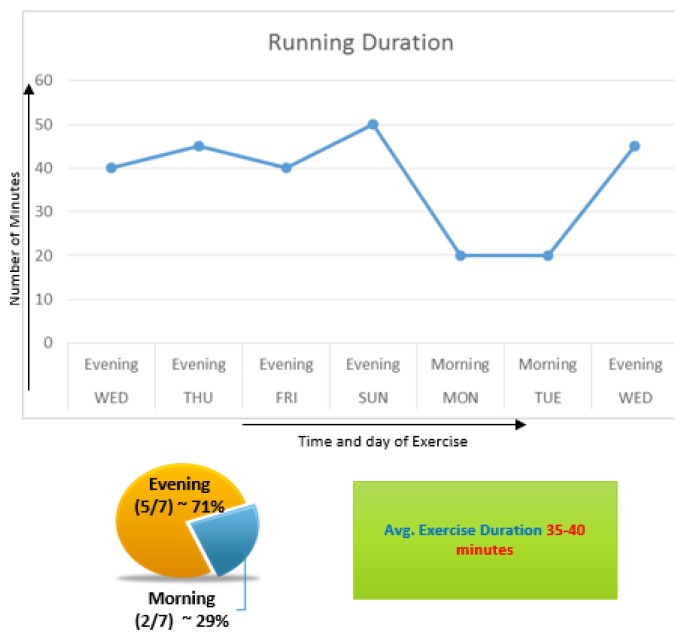
Visualization of favorite time during jogging.

Figure 5 shows a graph in which user running pattern is displayed. It shows how many minutes he jogged during each day and what part of day did he jog. Average jogging duration is displayed which can further help us determine the average calorie consumption per week. It shows that 71% of the running has taken place in the evening. This shows that the user is more comfortable jogging in the evening.

The behavior visualization is the pattern of the user in a prolonged period of time. The location of user's favorite restaurants can be retrieved and than it can be deduced what kind of food he is eating depending on the previous story. If he is eating fast food a lot it is reflected in the visualization. His preferred activity is also visualized so is his preferred restaurant, his preferred music and his favorite social network.

The daily activity component presents activity of users during different activities for each day as the timestamp is also sent to the cloud. This is similar to the life log visualization but it is more detailed for the most recent activity which is on the last day and the last week.

Resource management is one of the influencing factors in the mobile cloud computing due to constraints in the mobile device. The visualization of power usage of the mobile device relates to the information about the battery status of the mobile device. To track the consumption of the mobile application the battery consumption graph of the mobile device is also shown. This helps optimize the application and also indicate on which phase of execution, more battery was consumed. Moreover this information also gives the heads up about the battery depletion time when combined with the timestamp.

## 4. Behavior Life Style Analysis

All the components contribute to the behavior life style analysis which range from the activity recognition to context verification and context representation. We use the context information from different aspects of user's life like location, sensors, social media and environment. Associating different data sources and information and extracting a context from them is hard and the focus is trying to do it through behavior classification and correlating different patterns. The context of the data is analyzed from rule checking or ontology matching. If the data consists of ontological model than the data fetcher retrieves the specific pattern. If it is a rule then the pattern is checked by rule-based filtering algorithm. If the data consists ontological data model, then the match matching algorithm requests to the query generator module of the parser which generates the query according to the needs of the user, and then retrieve the data with the specific matched pattern.

The rule-based method requests to the rule-base repository via rules and checks the data based on rules. Once the pattern of the data has been matched, then the data is given to decision making in order to make the higher level decision based on the collected information. The decision making comes after the parsing and context analysis, the system gives suggestions against different activities. The activities are also visualized to have better understanding of the activities and behavior and take effective measures.

### Case Study

**Figure 6.**
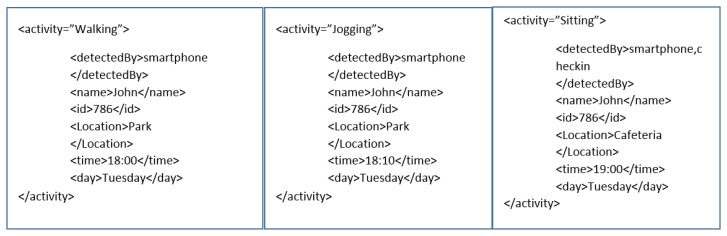
Context Information from activity recognition.

Figure 6 shows context information received by the behavior life style analysis. The first activity is walking around the park around 6 pm on a Tuesday. It means the user is back from work and taking a stroll in the park. Later in the second part the user is jogging 10 min later. This means he did a little exercise. At 7 pm the user is sitting in a cafeteria or restaurant. This indicate that he is going to have a meal. It also means that a certain amount of calories will be consumed. The food calories can be guessed from the restaurant check-in and user's life log history. From this an overall context can be extracted that the user calorie consumption is increased and he needs to be recommended a routine which will burn more calories. There can be a new rule generated against this routine which will be triggered in similar situations.

## 5. Implementation and Results

Experiments were done on a Hadoop cluster which consists of 4 host machines. Each host machine had four cores and 8 GB memory. 4 virtual machines were installed on two hosts which meant there are four virtual machines and 2 host machines. In total the cluster consisted of 6 nodes.

In Hadoop there are many traffic considerations as the nodes communicate a lot on the network during MapReduce jobs. There are different traffic types and job patterns as some jobs generates more intermediate data. The different types of traffic generated are admin related which consists of heart beats, time delay calculation and application messaging, then there is a data copying and replication which takes place in which there is a lot of traffic between the nodes. The data is compressed further to decrease the data that is being transferred and read by Hadoop. It decrease the network communication with a little tradeoff as Hadoop reads compressed data and has an additional task of uncompressing the data. One other Hadoop traffic type is intermediate data between map and reduce phase. In intermediate date when the output of mapper is released to be the input of the reducer in the shuffle phase. Splittable compression format is used which can be done on intermediate data in Hadoop and also the final output of the data.

An android application is developed for activity recognition. We used GPS and accelerometer, gyroscope, and proximity sensors. We used Samsung galaxy S3 for experiments. There are three different activities, *i.e.*, standing, walking and sitting for three different locations, *i.e.*, home, office and outdoor. There are six additional activities for outdoor locations, *i.e.*, jogging, rest in park, waiting at bus stop, having lunch in cafeteria, exercising in gym and riding a bus. These outdoor activities are very important in determining the user behavior as they play a huge part helping in finding the context of the situation.

For the evaluation and testing purpose, over 10,000 data samples were collected from 10 volunteer graduate students during a month-long period at various locations. Table 1 shows the minimum and maximum age, height and weight of the volunteers. After collecting sensor data from all volunteers, they were categorized into each activity types based on activity label.

Table 2 shows that in home location the average accuracy is 90.74% and around 9.26% error on average for the activities, *i.e.*, standing, walking and sitting. Similarly there is 9.68% of error when the user was standing. It can be seen that standing and sitting is very similar as the user tends to be stationary in these activities. Walking in home is one of the tricky part to identify as it is confused by both sitting and standing. It was identified 83.47% of the time and recognized as sitting and standing due to limited movement of user in house for walking. Sitting was the most accurate and had negligible confusion of 2.56% as walking.

Table 3 shows that in the office location the average accuracy is 95.91% and around 4.09% error on average for the activities, *i.e.*, standing, walking and sitting. The recognition in office is much accurate than the home activity recognition. Again the walking is little less accurate than sitting and walking but the results much better than the home accuracy. This is due to the bigger area and more movement which contributed to better accuracy. Sitting and standing also have some confusion due to similarity and less movement between these two activities.

In Table 4 for the outdoor location the average accuracy was 91.9% and around 8.1% error on average for the activities of standing, walking, waiting for a bus, having lunch at the cafeteria etc. The experiments were conducted on nine activities including five location-based activities (waiting for bus at bus stop, having a lunch at a cafeteria, exercising at gym, resting in the park). The location based activities are highly dependent on GPS. If the GPS is working correctly, the recognition process works very well.

**Figure 7.**
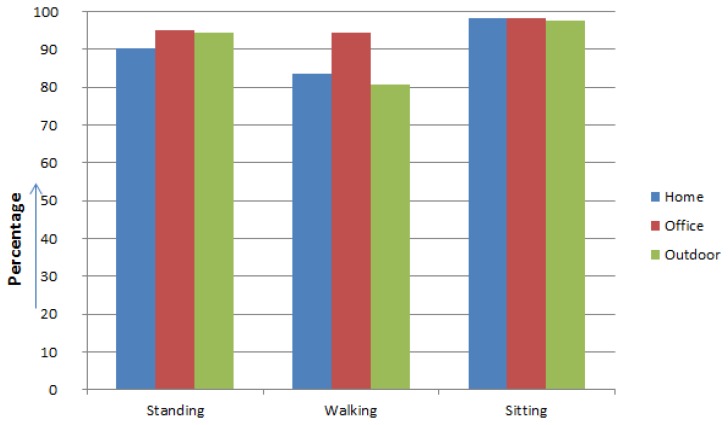
Activity Recognition accuracy in different locations.

There are many cases which show different accuracy for the same activities. This indicates that the activity can be recognized differently depending on what the location is. For example, walking activities in the home or outdoors are sometimes recognized as a standing given that the user frequently stops to change direction for house or office chores. The recognition accuracy of both sitting and standing activities are relatively higher than the others due to their static nature as shown in Figure 7.

### Monitoring Threshold

The battery consumption here is also presented in a graphical form. This provides an indication of how much battery is being consumed with one push of the data in the cloud. It is also indicative of whether some tweaking is required in the mobile application if the consumption is too high or may require a change in the settings of the mobile device.

**Figure 8.**
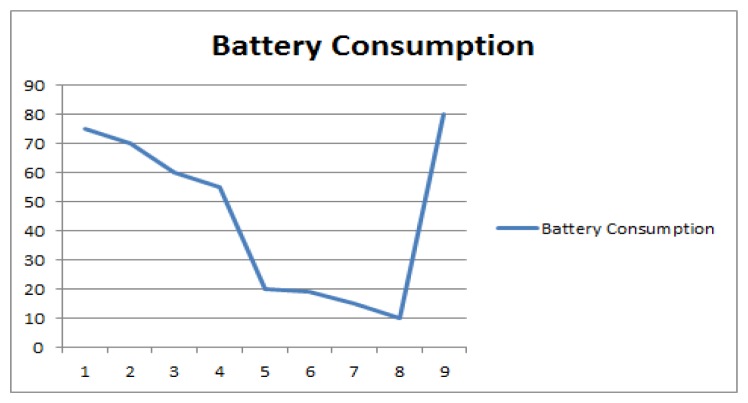
Battery Usage.

In Figure 8 a graph has been plotted with battery remaining in the mobile device when it sends the parameters. Here the mobile phone battery is constantly dropping. But there is sharp drop from around 55% of the battery to around 20%. This indicates that the resource consumption was very high at that point. A threshold can be defined when the battery life is 25% left, the application should stop monitoring the activities. Similarly at a particular threshold the application can stop using GPS for activity recognition. This affects the accuracy of the application and lower battery consumption ensures the longevity.

For sudden drops in battery, as all the messages have been time stamped we could check in the mobile application logs what operation was done at that time. This could help optimize the mobile application in terms of resource usage. The later jump to high battery time indicates that the mobile device has been charged.

## 6. Conclusions

In this paper a technique has been proposed for behavior life style analysis through activity recognition and large scale data mining in mobile sensory data through MapReduce. A mobile application is built which uses the embedded sensors in smartphones to recognize different activities in different locations like walking, standing, and sitting. All the data logs generated from the phone are transferred into the public cloud to further predict the lifestyle and behavior. Then Hadoop is used for extracting context from the data logs and use it for recommendations and suggestions. The application is energy aware as the battery time is also monitored as well as the resources used by the application and stop the application by a predefined threshold which results in longer battery life. Through different activities and location based mining effective behavior analysis can be done through semantic life log representation and making a timeline of events through visualization.

## Figures and Tables

**Table 1.** Participants of data set.

	**Minimum**	**Maximum**
Age	24	32
Height	170 cm	178 cm
Weight	132 lb	187 lb

**Table 2.** Activity Recognition Accuracy for Home.

**Activity at Home**	**Standing**	**Walking**	**Sitting**
Standing	90.32%	-	9.68%
Walking	10.43%	83.47%	6.1%
Sitting	2.56%	-	98.44%

**Table 3.** Activity Recognition Accuracy for Office.

**Activity at Home**	**Standing**	**Walking**	**Sitting**
Standing	95.5%	-	4.8%
Walking	4.84%	94.35%	0.81%
Sitting	1.2%	0.61%	98.19%

**Table 4.** Activity Recognition Accuracy for outdoor.

**Activity at Home**	**Standing**	**Walking**	**Sitting**	**Jogging**	**Riding Bus**	**Waiting For Bus**	**Have Lunch in Cafeteria**	**Exercise in Gym**	**Sit in Park**
Standing	94.34%	-	5.66%	-	-	-	-	-	-
Walking	12.77%	80.85%	6.38%	-	-	-	-	-	-
Sitting	2.5%	-	97.5%	-	-	-	-	-	-
Jogging	2.17%	10.86%	1.47%	85.5%	-	-	-	-	-
Riding Bus	16.25%	6.25%	1.25%	-	76.25%	-	-	-	-
Waiting For Bus	-	-	-	-	-	100%	-	-	-
Have Lunch in cafeteria	-	-	-	-	-	-	100%	-	-
Exercise in Gym	-	-	-	-	-	-	-	100%	-
Sit in Park	-	-	-	-	-	-	-	-	100%
